# Global population datasets overestimate flood exposure in Sweden

**DOI:** 10.1038/s41598-024-71330-5

**Published:** 2024-09-02

**Authors:** Konstantinos Karagiorgos, Stefanos Georganos, Sven Fuchs, Grigor Nika, Nikos Kavallaris, Tonje Grahn, Jan Haas, Lars Nyberg

**Affiliations:** 1 Risk and Environmental Studies, Karlstad University, Karlstad, Sweden; 2 Centre of Natural Hazards and Disaster Science (CNDS), Uppsala, Sweden; 3 Centre for Societal Risk Research (CSR), Karlstad University, Karlstad, Sweden; 4 Geomatics, Karlstad University, Karlstad, Sweden; 5 Department of Civil Engineering and Natural Hazards, BOKU University, Vienna, Austria; 6 Mathematics, Karlstad University, Karlstad, Sweden

**Keywords:** Flood exposure, Gridded population dataset, WorldPop, GHSPop, Flood risk management, Sweden, Environmental sciences, Natural hazards

## Abstract

Accurate population data is crucial for assessing exposure in disaster risk assessments. In recent years, there has been a significant increase in the development of spatially gridded population datasets. Despite these datasets often using similar input data to derive population figures, notable differences arise when comparing them with direct ground-level observations. This study evaluates the precision and accuracy of flood exposure assessments using both known and generated gridded population datasets in Sweden. Specifically focusing on WorldPop and GHSPop, we compare these datasets against official national statistics at a 100 m grid cell resolution to assess their reliability in flood exposure analyses. Our objectives include quantifying the reliability of these datasets and examining the impact of data aggregation on estimated flood exposure across different administrative levels. The analysis reveals significant discrepancies in flood exposure estimates, underscoring the challenges associated with relying on generated gridded population data for precise flood risk assessments. Our findings emphasize the importance of careful dataset selection and highlight the potential for overestimation in flood risk analysis. This emphasises the critical need for validations against ground population data to ensure accurate flood risk management strategies.

## Introduction

Flooding is a global challenge that affects many regions worldwide. Over the past 20 years, more than 1.6 billion people have been impacted globally, with estimated losses surpassing 1 trillion US dollars^1^. Flood impacts can be mitigated through flood risk management, and high-risk areas can be identified by flood risk assessments^2^. These assessments rely on the conceptual framework that considers flood risk as a combination of hazard (the flood event), exposure (the people and assets at risk), and vulnerability (the susceptibility of the exposed elements)^3^. While significant efforts have been made in developing hazard and vulnerability assessments, the assessment of exposure still remains fragmentary in many practical applications^4^ and is thus under-researched^5,6^.

Apart from other elements at risk, population information plays a critical role in supporting exposure assessments^7^. The availability of such information, however, is often limited particularly in countries with less detailed or infrequent censuses^8,9^ or due to confidentiality, privacy issues and nondisclosure requirements^4^. As a result, high-resolution census population data are mostly classified, and national census authorities typically provide such data at pre-defined and aggregated spatial resolution or statistical division^10^. To overcome this gap, available digital spatial data, such as those derived from multi-temporal high-resolution satellite imagery has been used to derive gridded population data that can be used in flood risk assessments. As a consequence, exposure analysis focusing on population have been conducted at different scales^2,11,12^ using such gridded data (see Smith^13^ for further discussion), but results have hardly been critically evaluated so far^4^.

The scientific community has increasingly demonstrated how to create global georeferenced data to address the information gaps in low-income countries^14^ or to overcome inconsistencies in census-derived national population data^8^. Over the past 25 years, the number of available gridded population datasets has grown significantly. Gridded population mapping involves allocating census data to spatial units (grid cells) of a specific size based on a population distribution model^15^. Some of the most widely used gridded population products include the WorldPop database^16^, the Global Human Settlement Layer Population (GHSPop)^17^, the Gridded Population of the World (GPW)^18^, the Global Rural Urban Mapping Project (GRUMP)^19^, the Landscan population database^20^ and the High Resolution Settlement Layer (HRSL)^21^. These datasets are applied in a wide variety of research areas, enhancing evidence-based decision-making. However, several studies using these datasets often neglect to justify their choice of dataset, even though it has been demonstrated that the selection of data can significantly impact the outcomes^16,22^.

Even though the gridded population products utilize comparable input data (census data, administrative boundary data and geospatial correlates), there are notable discrepancies when compared to ground observations^23^. To evaluate the reliability of gridded population datasets, several comparative analyses have been conducted^22–27^. The majority of these focus on total population estimates, with very few studies aiming to quantify population exposure to various natural hazards^28–30^ and even fewer specifically addressing flood exposure^4,10,13^. Additionally, current studies validating flood exposure have been limited to using either a 1 km grid or relying on synthetic data^4,13^. Uncertainty is inherent in population estimates and there is currently no accepted method to quantify or communicate the level of uncertainty associated with the available data products^31^. While differences in population counts for most counties are insignificant, they can be significant in smaller administrative units^32^. Objective comparisons can support our understanding of the differences and limitations of the various datasets and the nature of these differences. Population grids ultimately need to be validated against ground population data to ensure the most accurate estimates^31^. Furthermore, a challenge faced by all the producers of gridded population estimates is the lack of spatially detailed datasets that correlate with the variation of population density across small areas. Accurate fine-scale gridded population data is needed for these datasets to be useful in policy and practice^33^.

While gridded products are becoming integral to decision-making processes for various stakeholders, the discussion of the fitness for use of spatial data, particularly concerning scale, has received less attention^8^. Users of gridded products often attempt to model a specific process of interest, but there is frequently a mismatch between the operational scale and the analytical scale^8^. Although gridded products offer high-resolution estimates, this does not inherently ensure greater accuracy at the analytical scale. In fact, uncertainties and errors tend to escalate as the resolution increases^31^. These effects are described in the literature as the Modifiable Areal Unit Problem (MAUP)^34^. MAUP is a potential source of error in generated population studies; however, most of these studies overlook its impact on their results^15^.

The aim of this study is to contribute to the ongoing discussion regarding the accuracy and suitability of gridded population datasets for flood exposure analyses. This is accomplished by evaluating the discrepancies between two commonly used gridded population datasets and official population statistics in Sweden at a 100 m grid cell level. The first objective is to quantify the flood exposure reliability of two globally available gridded population datasets (WorldPop and GHSPop) by comparing them to a national reference dataset in Sweden. Although other datasets exist, they were excluded from this analysis due to differences in spatial resolution (not available at the 100 m grid cell level), temporal limitations (not available for the specific year of analysis), unavailability of data in the study area and lack of global coverage. The second objective is to assess and quantify the impact of data aggregation on estimated flood exposure at different administrative levels.

## Results

### Flood exposed population

**Fig. 1:**
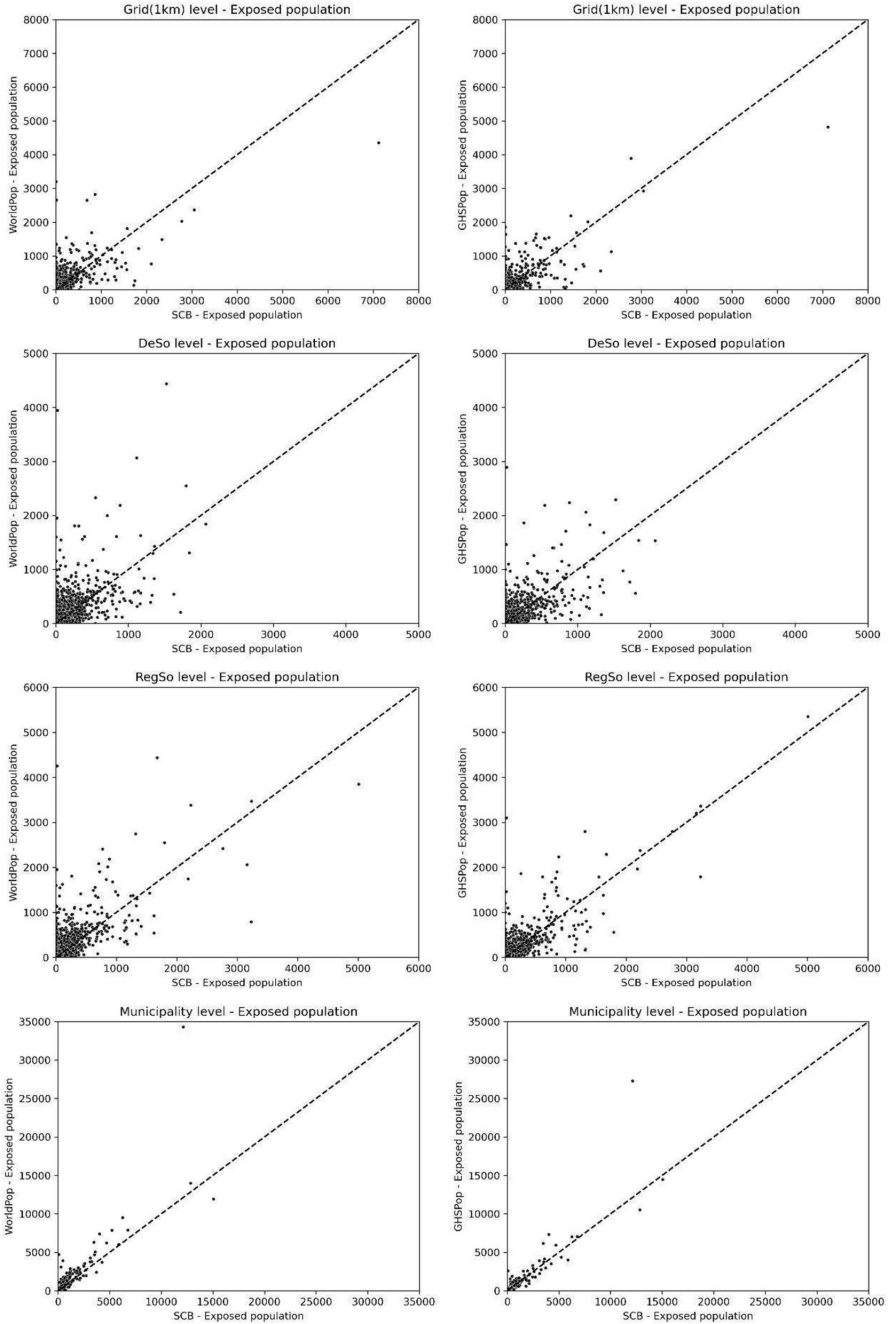
Scatter plots comparing the exposed population estimates from the reference dataset (SCB—Swedish Statistical Bureau) to those from the generated datasets, WorldPop (left column) and GHSPop (right column), across four administrative levels : Grid (1 km), Demographic Statistical Areas (DeSo), Regional Statistical Areas (RegSo), and Municipality. Each plot shows the SCB estimates on the x-axis and the corresponding estimates from WorldPop and GHSPop on the y-axes. The dashed line represents the 1:1 ratio, indicating perfect agreement between the datasets. The dispersion of points around this line illustrates the degree of correlation and potential discrepancies in population exposure estimates between the different sources and administrative divisions.

Table 1 presents a statistical analysis comparing flood exposure estimates derived from the reference population data provided by the Swedish Statistical Bureau (SCB) with those extrapolated using modelled populations generated by WorldPop and GHSPop across various administrative divisions. The analysis reveals that the GHSPop model generally outperforms the WorldPop model in nearly all metrics, exhibiting a discernible linear trend (see the bottom two scatter plots in Fig. 1). At the municipal level, the results are particularly reliable for both modelled datasets. The GHSPop model accounts for approximately 80% of the variability (R^2^ = 0.80), while the WorldPop model accounts for about 74% of the variability (R^2^ = 0.74), with few outliers. Additionally, the Root Mean Square Error (RMSE) at the municipal level indicates that GHSPop data significantly surpasses WorldPop data (GHSPop RMSE = 1003 versus WorldPop RMSE = 1484), as shown in Table 1. As the analysis progresses to regional (RegSo), demographic (DeSo), and 1 km grid areas, the model’s reliability diminishes, and the linear trends dissipate. When comparing the two modelled populations with the reference data using Mean Absolute Error (MAE), GHSPop consistently outperforms WorldPop, with the exception of the 1 km grid level.

**Table 1:** Performance metrics of population comparison from the reference dataset (SCB—Swedish Statistical Bureau) to those from the generated datasets at different administrative boundary levels, using WorldPop and GHSPop datasets.

	Administrative boundaries	n	R-squared	RMSE	MAPE	%MAE
WorldPop	Grid (1 km)	534,712	0.49	13	118.42	73.41
	DeSo	5985	0.41	140	121.85	67.68
	RegSo	3363	0.53	191	115.17	61.57
	Municipality	290	0.74	1484	47.34	38.13
GHSPop	Grid (1 km)	534,712	0.63	11	168.87	74.07
	DeSo	5985	0.50	105	149.91	65.2
	RegSo	3363	0.67	138	126.73	55.66
	Municipality	290	0.80	1003	43.18	31.32

The administrative levels include Grid (1 km), Demographic Statistical Areas (DeSo), Regional Statistical Areas (RegSo), and Municipality. The performance is evaluated based on R-squared, Root Mean Squared Error (RMSE), Mean Absolute Error (MAPE) and Percentage Mean Absolute Error (%MAE).

Figure 1 presents scatter plots comparing flood exposure estimates based on known population data from SCB with those derived using generated population data from WorldPop and GHSPop across different administrative levels: Grid, DeSo, RegSo and Municipality. The scatter plots reveal a positive correlation at all administrative levels; however, the strength of this correlation varies significantly, with some levels displaying tighter clustering and others showing greater dispersion.

At the grid level, there is a discernible trend between the known population dataset and the generated datasets, albeit with some scatter, indicating variability in the accuracy of WorldPop and GHS-Pop compared to the SCB dataset. Moving to the DeSo level, the scatter tightens, especially noticeable for WorldPop, suggesting better consistency at this administrative level. Conversely, at the RegSo level, the points exhibit wider dispersion, particularly for the GHSPop dataset, indicating less consistency in population estimates. Finally, at the municipality level, both WorldPop and GHSPop datasets show a tighter cluster of points, with GHSPop demonstrating fewer outliers and thus higher accuracy at this level.

**Fig. 2:**
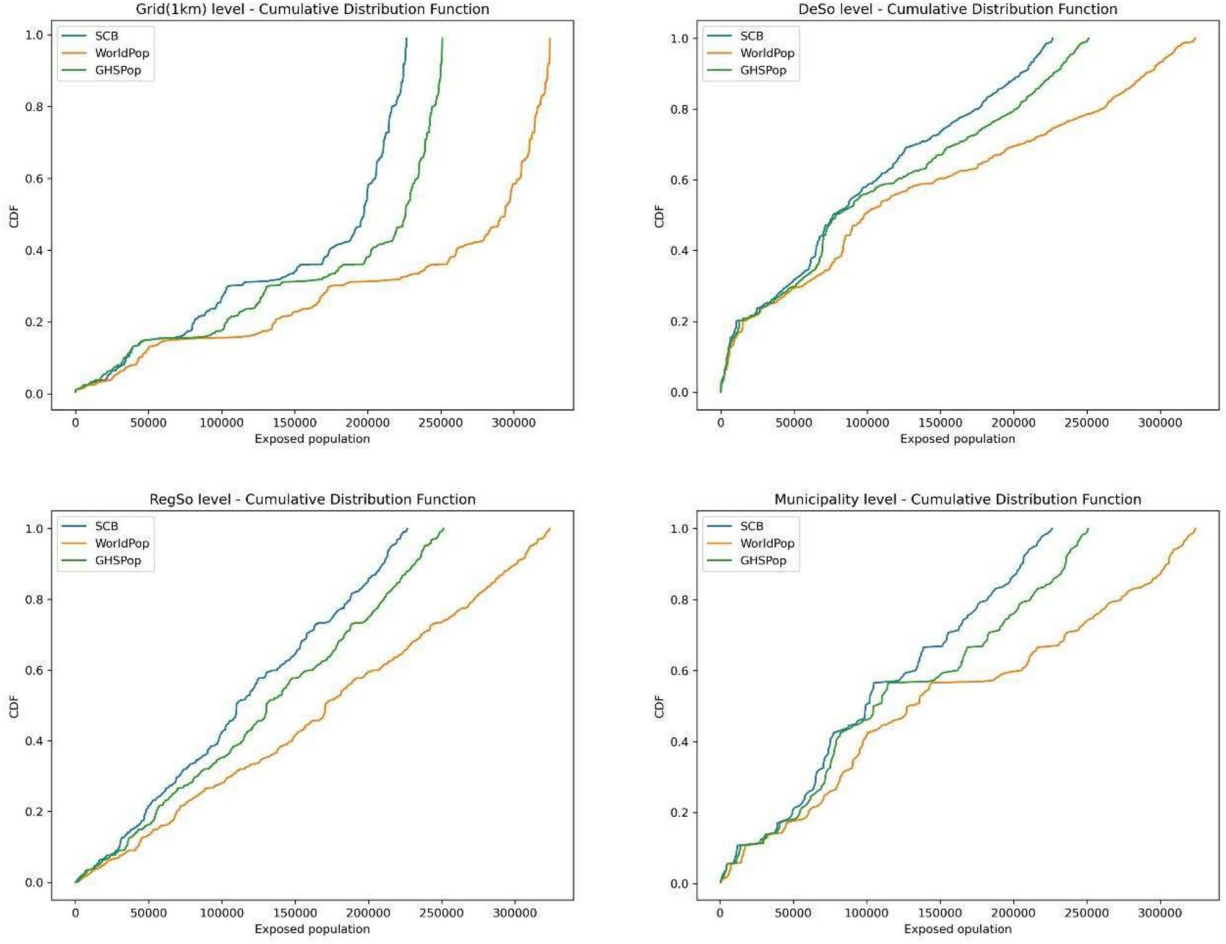
Cumulative Distribution Functions (CDFs) comparing the exposed population estimates from the reference dataset (SCB—Swedish Statistical Bureau) to those from the generated datasets, WorldPop and GHSPop , across four administrative levels : Grid (1 km) (n = 534712), Demographic Statistical Areas (DeSo) (n = 5985), Regional Statistical Areas (RegSo) (n = 3363), and Municipality (n = 290). Each plot shows the CDF of exposed population estimates, with SCB in blue, WorldPop in red and GHSPop in green. The x-axis represents the exposed population, while the y-axis represents the cumulative probability.

Figure 2 depicts the cumulative distribution functions (CDFs) of flood exposure estimates based on known population data from SCB, compared with those derived using generated population data by WorldPop and GHSPop across various administrative boundaries. In all four graphs, both WorldPop and GHSPop datasets show a similar overall trend in their estimates across the population range. However, both datasets consistently tend to overestimate the exposed population. WorldPop overestimates the exposed population by 35%, while GHSPop overestimates it by 10%. Across all administrative boundaries, WorldPop consistently estimates higher numbers of individuals in flood zones and the discrepancy between the three datasets increases with population size. At the grid and RegSo levels, the estimates are comparable in the lower population ranges, but as population size increases, both datasets significantly overestimate the exposed population. At the DeSo and municipality levels, estimates are similar for low and medium population ranges, but overestimation occurs in higher population ranges.

**Fig. 3:**
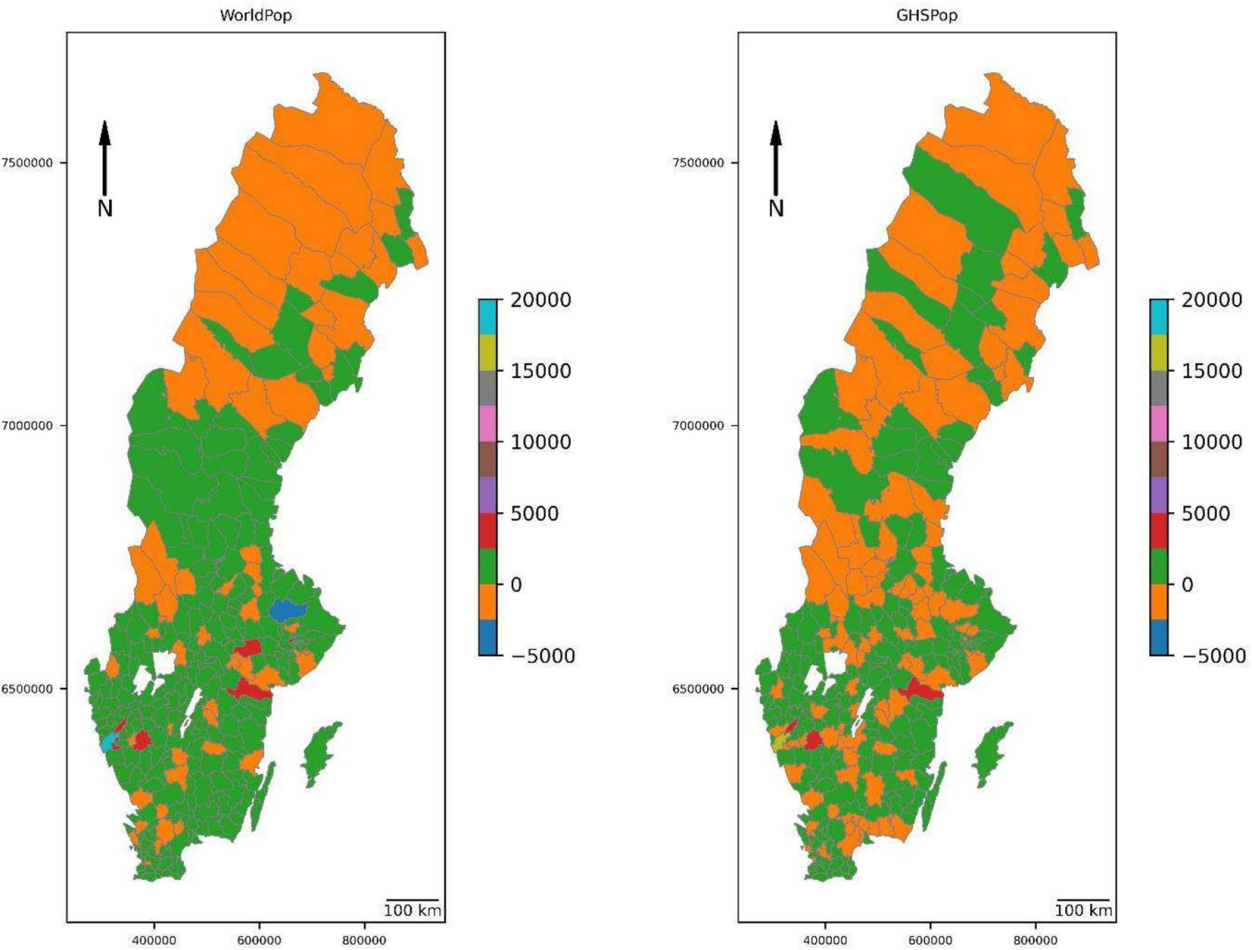
Differences in exposed population estimates between the reference dataset (SCB—Swedish Statistical Bureau) to those from the generated datasets, WorldPop and GHSPop at the municipality level.

**Fig. 4:**
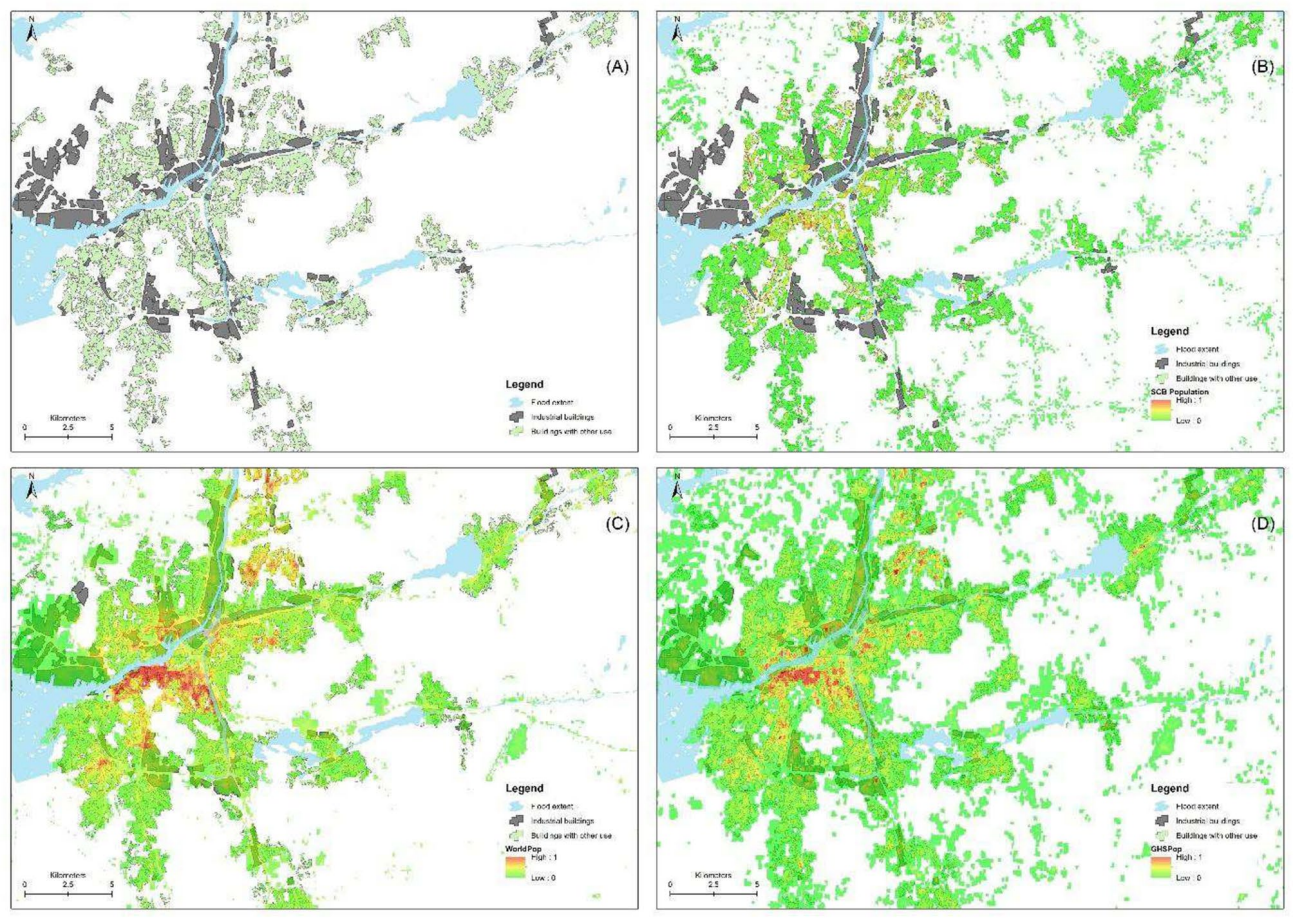
Comparison of population distribution between the known population data from SCB (**B**) with those derived using generated population data by WorldPop (**C**) and GHSPop (**D**) at Gothenburg municipality, considering flood extent and building uses (**A**).

Figure 3 demonstrates the differences in population flood exposure at the municipal level, contrasting known population data from SCB with estimates derived from generated population data by WorldPop and GHSPop. Both models show underestimation in northern and central Sweden, regions primarily rural with lower population densities. In contrast, overestimation is evident in major cities and suburban areas known for extensive industrial and commercial activities. The most substantial overestimation occurs in Gothenburg municipality for both datasets. To explore this discrepancy, we compared information related to population densities across the three datasets and examined building usage in the area (Fig. 4). In Gothenburg, industrial buildings (Fig. 4A) are primarily clustered along major waterways and transportation routes. There is also significant industrial presence close to central urban space, with smaller clusters distributed in peripheral regions. The SCB reference dataset (Fig. 4B) shows high population density areas concentrated around central urban areas, gradually decreasing towards rural peripheries. In contrast, the WorldPop generated population distribution (Fig. 4C) suggests higher population densities in urban areas than observed in reality, with notable overestimation in specific industrial zones. Similarly, the GHSPop dataset (Fig. 4D) indicates overestimation in both central and peripheral urban areas.

## Discussion

Population data are essential components in risk assessment and management. Due to privacy concerns and the unavailability of high-resolution census population datasets, many disaster studies rely on modelled global population datasets at finer spatial resolutions. However, the accuracy and suitability of these datasets has to be carefully evaluated. In the age of open data, it is crucial to assess the appropriateness of a dataset and to quantify potential uncertainties. Our national-scale comparison of flood exposure represents a significant advancement over previous studies, which repeatedly conducted validations only at a kilometer grid level. In this study, two gridded population datasets—WorldPop and GHSPop—were evaluated in the context of flood exposure. Equally important, the impact of data aggregation at different administrative levels was also assessed and quantified.

Based on the statistical comparisons conducted, it was found that GHSPop’s population estimates outperform WorldPop’s across nearly all the metrics when assessing flood exposure. So far, comparisons at a 100 m grid resolution for flood exposure have not been previously documented in the literature. In studies comparing coarser resolutions, Tuholske et al.^35^ examined five gridded population datasets (GPW-15, GHSPop, WorldPop, Landscan and ESRI World Population Estimates) to estimate the proportion of population residing in flood-prone areas. They observed significant variations in estimates of exposed population at 1 km × 1 km resolution across different products. Notably, GHSPop provided more accurate estimates compared to the WorldPop-Global-Unconstrained dataset. In another comparison of four global datasets, Mohanty and Simonovic^4^ concluded that WorldPop performs better than GHSPop in a 1 km × 1 km comparison.

Comparison studies of gridded population datasets reveal significant variation in precision across different scales and locations. This variability in accuracy can lead to diverse conclusions and decisions depending on the dataset chosen for analysis^36^. The findings from this study underscore the necessity of validating global population datasets against fine-resolution reference datasets to achieve the most accurate estimates. Users must carefully evaluate and comprehend the characteristics of different population datasets to select the most suitable option. Moreover, fine-scale validations could offer crucial insights and enhancements to the modelling methods and inputs used in these datasets.

Interestingly, both datasets under evaluation demonstrate optimal performance at the municipal level. The performance metrics are as expected, confirming quantitatively that despite employing different methods to construct the gridded population datasets, both datasets align closely with census counts at the administrative unit level, adjusted to correspond with UN estimates. Consistent with prior research^37–39^, our findings indicate a decline in predictive accuracy as the model shifts to finer administrative levels. One contributing factor to these discrepancies at finer spatial scales may be the absence of detailed land-use information in dasymetric models. This includes distinctions between residential and non-residential built-up areas, as well as the incorporation of relevant predictors such as building volume and elevation. Additionally, population distribution is non-random, which means that how population is allocated and represented will always be influenced by aggregation effects. According to Leyk et al.^8^, it is crucial to acknowledge that the MAUP significantly impacts the suitability of data products in analyses where precise spatial positioning of population is essential. This finding aligns with research by other scholars investigating exposure to natural hazards, such as Fuchs et al.^40^. The use of finer resolution data in this study underscores the importance of ongoing testing of gridded population products across various spatial resolutions. While users may naturally prefer the highest resolution population data available, they should carefully assess whether this effectively meets their specific needs^31^.

The analysis conducted in this study reveals significant overestimates in the exposed population to flooding when comparing official known population data with generated population datasets at a national scale. Both evaluated datasets, WorldPop and GHSPop, consistently overestimated the exposed population at national level across various administrative units examined. The findings contradict previous studies, which suggested that generated gridded population datasets tend to underestimate exposed populations. For example, Mohanty and Simonovic^4^ assessed census-level population data from Statistics Canada alongside four generated datasets at 1 km × 1 km resolution, finding that all global population datasets underestimated the actual population. When comparing the different generated population datasets in this study, it was observed that flood exposure estimates using GHSPop resulted in lower overestimates compared to WorldPop. This contrasts with Mohanty and Simonovic findings^4^, where WorldPop provided estimates closest to the official Canada census data, followed by LandScan, GPW, and GHSPop. Importantly, our analysis utilizes a finer 100 m x 100 m official population dataset, whereas Mohanty and Simonovic^4^ used a coarser 1 km x 1 km official population grid. This highlights that the spatial scale of evaluating population data can introduce uncertainties, with finer resolutions potentially reducing them. Given that flooding is a highly localized phenomenon using coarser resolutions of population can pose challenges. This aligns with Smith et al.’s^13^ conclusion that combining high-resolution population data with high-resolution hazard data leads to more accurate exposure assessments.

Given the significant overestimates, a challenge encountered by producers of gridded population data is the scarcity of spatially detailed datasets that adequately reflect population density across small intra-urban areas^33^. Although geospatial covariates are used to correlate the presence or absence of people, none of these datasets is reflective of the locations of high concentrations. Random Forest models utilize covariates such as land cover types and night-time lights, which typically have a resolution coarser than 100 m × 100 m. This lead to a “halo” effect, where population is assigned to cell adjacent to settlements rather than directly over them^36^. It is crucial that the next versions of population distributions maps constrain their disaggregation within high-quality, accurate building footprint layers that are becoming increasingly available^41,42^. Additionally, the example of Gothenburg demonstrates how land use and the presence of industrial buildings can significantly influence the distribution of population and underscore the discrepancies between real and modeled population data particularly in distinguishing between residential and non-residential land uses. These findings suggest areas of improvement in the population models and more work will be needed to develop accurate datasets for the distinction of land use to avoid population being misallocated to industrial districts, universities, airports and other non-residential cells^36,43,44^.

Flood risk assessment and management are major applications for gridded population data, if exposure has to be evaluated. The primary objective of the study was to compare the population estimates from different datasets in the context of flood exposure. The consistent methodology applied across all datasets ensures that the comparison is valid and any observed discrepancies are due to differences in the datasets rather than the method of analysis. The analysis presented in this study provides valuable insights for users of global gridded population products. It offers a quantitative comparison between known and two generated population datasets, clearly illustrating the differences among them. This study, although focused on Sweden, presents findings with broad implications for stakeholders utilising large-scale flood exposure data in risk analysis and decision-making processes. We recommend that researchers and decision makers acknowledge the inherent uncertainty associated with these products. To better characterise this uncertainty, users should incorporate multiple grids in their analyses instead of relying solely on a single data product. Our findings underscore the need for further validation research and thorough scrutiny of gridded population datasets. Future studies should prioritize cross-country evaluations, as emphasized in existing literature^31^ which calls for a systematic global comparison rather than focusing solely on individual countries. Our aim is to advance these findings by examining more detailed population datasets, such as High-Resolution Settlement Layer (HRSL) and employing dasymetric techniques at the individual building level^45^.

## Data and methods

### Data

#### Official national population dataset

As the reference for the known population, the total population of Sweden represented in a 100 m × 100 m vector grid has been used. The dataset is made available by the Swedish Statistical Bureau (SCB), where the input information is based on the Swedish population register. The Swedish population register includes all the registered residents in Sweden both Swedish citizens and non-Swedish citizens with a residence permit for a minimum of 12 months. To generate the grid data each individual in the population register is geocoded to their specific residence location and this information is then generalized to the grid code, based on the centroid of each residential building. This data is available exclusively for research purposes and can be accessed upon special request to SCB.

#### Generated population datasets

##### WorldPop

WorldPop provides open-access to gridded demographic indicators. The dataset was developed by the WorldPop project and is available at https://www.worldpop.org/. In our case, we used the 2020 constrained population product of population counts at approximately 100 m spatial resolution in the world geodetic system WGS84. To re-allocate population counts into gridded pixels, a semi-automated, dasymetric approach that incorporates census and ancillary data is used, employing a random forest estimation technique. The ancillary spatial data include settlement locations, settlement extents, land cover, roads, building maps, health facility locations, satellite nightlights, vegetation, topography, and refugee camps^46^. The constrained product restricts the population disaggregation only within built-up areas. Naturally, these data can vary from country to country based on data availability. Moreover, the generated gridded population datasets have been adjusted to match the United Nations’ population estimates.

##### GHSPop

The GHSPop dataset provides residential population estimates at approximately 100 m spatial resolution in the Mollweide projection and the WGS84 reference systems. The dataset was developed by the Joint Research Center (JRC) within the Global Human Settlement Layer (GHSL) project and is available at https://ghsl.jrc.ec.europa.eu/download.php?ds=pop. It covers the period from 1975 to 2030 in 5-years intervals. The fundamental inputs encompass vector-based population estimates provided by the *Center for International Earth Science Information Network* (CIESIN) for the *Gridded Population of the World* (GPWv4.11) at polygon level. These estimates are disaggregated from census or administrative units to grid cells, informed by the distribution, classification, and volume of built-up as mapped in the GHSL global layers for each corresponding epoch, produced from Landsat imagery collections. To improve accuracy, the generated gridded population datasets are rescaled to match the total population time series at ’city’ level from the extended database feeding the UN World Urbanization Prospects 2018, and the total population time series at country level provided by the UN World Population Prospects 2022^47^.

#### Administrative divisions

**Fig. 5:**
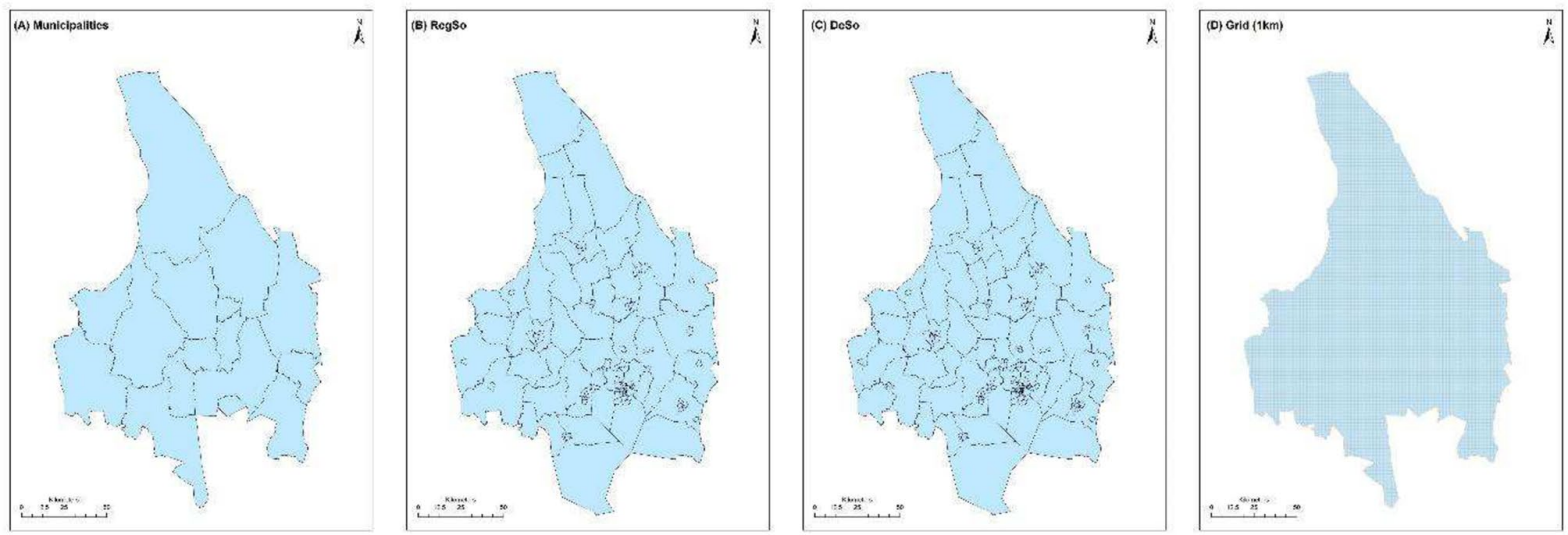
An example (Värmland county) of the different administrative units in Sweden. Municipalities (**A**), Regional Statistical Areas (RegSo) (**B**), Demographic Statistical Areas (DeSo) (**C**) and 1 km grid (**D**) division.

The administrative divisions used in this study consist of four levels: the 1 km grid, Demographic Statistical Areas (DeSo), Regional Statistical Areas (RegSo), and municipal levels (Fig. 5). The 1 km grid provides national coverage. The DeSo level, comprising 5985 areas, each with a population between 700 and 2700 inhabitants, and represents a nation-wide breakdown along county and municipal boundaries. DeSo areas tend to be stable and do not change over time. However, there is an exception: these areas might be subdivided in the future if their population composition and urban boundaries in particular change significantly. Similarly, the RegSo level is encompassing 3363 areas, each with a population ranging from 663 to 22,622 inhabitants, and represents a nation-wide breakdown along county and municipal boundaries. RegSo areas are stable and do not change over time unless there are any alterations to the county or municipal divisions, in which case the RegSo boundaries will be adjusted accordingly. Lastly, at the municipal level, there are data available for 290 municipalities. All the aforementioned datasets are freely available and can be accessed via SCB.

#### Flood hazard dataset

##### Swedish Civil Contingencies Agency (MSB) open access 100-year floodplain data

To estimate the number of people exposed to river flooding, the spatial distribution of flood hazards is represented by a 100-year flow, developed by the *Swedish Civil Contingencies Agency* (MSB) according to the requirements of the *European Flood Risk Directive* (Directive 2007/60/EU). These datasets serve as the national standard employed by MSB and the county administrative boards for the development of flood risk management plans. The MSB flood data are stored as polygons in ESRI Shapefile format and are freely available for download from the MSB’s flood portal (https://gisapp.msb.se/Apps/oversvamningsportal/index.html).

### Methods

#### Geospatial methods

The SCB population data are presented in a vector polygon format, while WorldPop and GHSPop are in raster format. The raster datasets were converted into a vector format and re-projected to match the coordinate system (SWEREF99) of the known population data.

Regarding the methodology applied in this study, we have defined *exposure* as the intersection of hazard and population data. An evaluation of the total population ensued, aligning the acknowledged total population by the SCB with the aggregate populations derived from WorldPop and GHSPop datasets.

To estimate the number of people exposed to river flooding, MSB hazard data, described as inundation areas, were intersected with population grids from both the known and generated datasets: SCB, WorldPop and GHSPop. Firstly, the intersection with the hazard data involved selecting population squares (100 m resolution) from both the known and generated population datasets that overlap with the inundated areas. Secondly, these inundated cells were converted into discrete points by calculating their centroids to facilitate a spatial join. This transformation was performed to optimize computational efficiency and prevent polygon double counting across two administrative levels. With this approach, we ensure that a population cell is only counted as inundated if the centroid of the area has been identified as being affected by the respective inundation polygon. Finally, a spatial join was performed to achieve a cohesive aggregation, allowing for the calculation of exposed population figures for each unit across various administrative levels, including the 1 km grid, Demographic Statistical Areas (DeSo), Regional Statistical Areas (RegSo), and municipal levels.

To evaluate the analyses developed for the various administrative boundaries based on known and generated populations, comparison statistics were calculated. The statistic metrics used included the Root Mean Squared Error (RMSE) as shown in Eq. (1), the Mean Absolute Percentage Error (MAPE), as shown in Eq. (2) and the percent Mean Absolute Error (%MAE), as shown in Eq. (3).

1 $$RMSE = \sqrt {\frac{1}{n}\mathop \sum \limits_{i = 1}^{n} \left( {y_{i} - \hat{y}_{i} } \right)^{2} }$$

2 $$MAPE = \frac{100\% }{n}\mathop \sum \limits_{i = 1}^{n} \left| {\frac{{y_{i} - \hat{y}_{i} }}{{y_{i} }}} \right|$$

3 $$\% MAE = \frac{1}{n}\mathop \sum \limits_{i = 1}^{n} \left| {y_{i} - \hat{y}_{i} } \right| \times 100\%$$

The variable ${y}_{i}$ indicates the known exposed population of sample $i$ from the official population data, and $\hat{y}_{i}$ indicates the generated exposed population of sample $i$ from the population data for the two gridded datasets. RMSE represents the square root of the average squared difference between the actual and synthetically generated population values. Among the three metrics, we prioritize the MAE due to its heightened resilience in the presence of outliers. While RMSE (linked with the value of R^2^, which represents the proportion of the variance for the dependent variable that is explained by an independent variable in the model) stands as a conventional statistical metric, it accentuates larger errors disproportionately due to the squaring of values. Additionally, in contrast to RMSE, both MAE and MAPE offer a straightforward interpretation between the observed and predicted values. In the last step, Cumulative Distribution Functions (CDFs) were developed to understand characteristics of the exposed population distribution across the three datasets at various administrative levels. By examining the shapes of CDFs, important characteristics of the distribution were extracted, such as the concentration of the data, its dispersion, and overestimations and underestimations.

## Data Availability

The official national population dataset used in this study, provided by Statisitska centralbyrån (SCB), is available exclusively for research purposes and can be accessed upon special request to SCB (https://www.scb.se/). The WorldPop dataset, developed by the WorldPop project, is publicly available at https://www.worldpop.org/. The GHSPop dataset, developed by the Joint Research Center (JRC) within the Global Human Settlement Layer (GHSL) project, is publicly available at https://ghsl.jrc.ec.europa.eu/download.php?ds =pop. Administrative divisions are freely available and can be accessed via SCB (https://www.scb.se/). The flood hazard dataset, developed by the Swedish Civil Contingencies Agency (MSB), is freely available for download from the MSB’s flood portal (https://gisapp.msb.se/Apps/oversvamningsportal/index.html).
